# Tuberculosis of Bones and Joints

**Published:** 1893-01

**Authors:** 


### BOOK NOTICES.

Tuberculosis of Bones and Joints. By N. Senn, M. D., Ph. D., Professor of Practice and Surgery in Rush Medical College. Illustrated with 107 engravings (seven of them colored). In one handsome royal octavo volume. 520 pages. Extra cloth, $4.00 net; sheep, $5.00 net; half Russia, $5.00 net. Philadelphia: The F. A. Davis Co., Publishers, 1231 Filbert Street.

The object of the author in writing this book has been to collect from recent literature the modern ideas on tubercular disease of the bones and joints, with the results of his own experience. Tuberculosis of the bones and joints is such a common affection that many, many cases present themselves to the general practitioner. The successful treatment of these affections depend on an early and correct diagnosis and the adoption of a timely and rational treatment of the disease. The author gives a history of the disease, the proofs of the disease, and the treatment of tuberculosis of the joints. The treatment is well illustrated by the many illustrations that are found throughout the work, which on the whole will prove beneficial to the student and physician. We certainly agree with him on the necessity of an early and correct diagnosis resulting in a successful cure, thereby saving the patient from a long life of misery.

